# Activity Map and Transition Pathways of G Protein-Coupled Receptor Revealed by Machine Learning

**DOI:** 10.1021/acs.jcim.3c00032

**Published:** 2023-04-10

**Authors:** Parisa Mollaei, Amir Barati Farimani

**Affiliations:** † Department of Mechanical Engineering, Carnegie Mellon University, Pittsburgh, Pennsylvania 15213, United States; ‡ Department of Biomedical Engineering, Carnegie Mellon University, Pittsburgh, Pennsylvania 15213, United States; § Machine Learning Department, Carnegie Mellon University, Pittsburgh, Pennsylvania 15213, United States

## Abstract

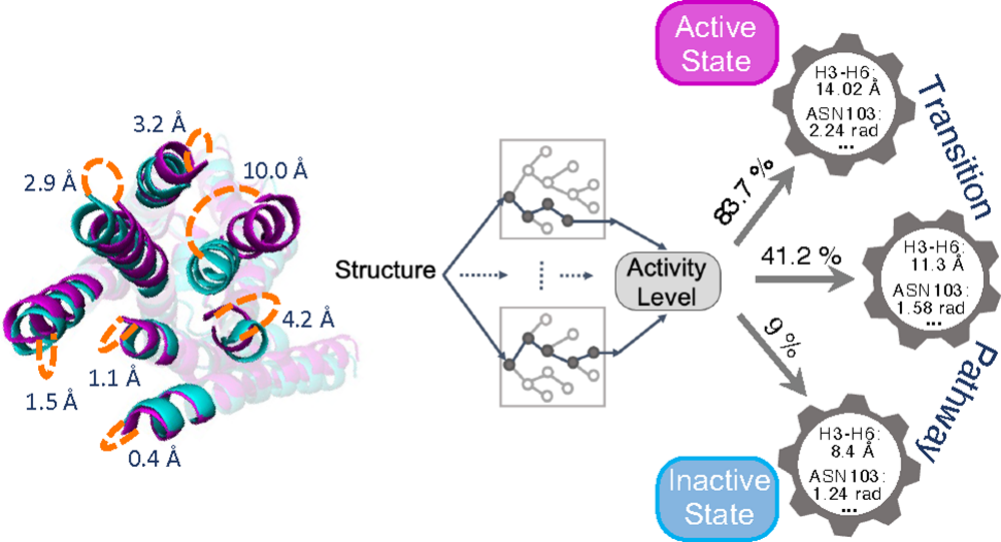

Approximately, one-third of all U.S. Food and Drug Administration approved drugs target G protein-coupled receptors (GPCRs). However, more knowledge of protein structure–activity correlation is required to improve the efficacy of the drugs targeting GPCRs. In this study, we developed a machine learning model to predict the activation state and activity level of the receptors with high prediction accuracy. Furthermore, we applied this model to thousands of molecular dynamics trajectories to correlate residue-level conformational changes of a GPCR to its activity level. Finally, the most probable transition pathway between activation states of a receptor can be identified using the state-activity information. In addition, with this model, we can associate the contribution of each amino acid to the activation process. Using this method, we can design drugs that mainly target principal amino acids driving the transition between activation states of GPCRs. Our advanced method is generalizable to all GPCR classes and provides mechanistic insight into the activation mechanism in the receptors.

## Introduction

The G protein-coupled receptors (GPCRs) are known as cell-surface receptors mediating approximately two-thirds of human hormones and one-third of the U.S. Food and Drug Administration (FDA) approved drugs.^1−8^ The GPCRs play a crucial role in the signaling cascade inside a cell making them essential targets in drug design and molecule-based therapeutics,^2,9^ such as in cancer therapy.^8,10^ However, currently, only about 25% of potential druggable GPCRs are being targeted in the drugs due to insufficient experimental and computational knowledge about the mechanisms of conformational changes in the receptors.^11^ The GPCRs agonists are remarkably diverse including photons, odorants, viruses, vitamins, peptides, and nonpeptidergic hormones.^12−14^ Once a ligand binds to the extracellular part of a receptor, it triggers some conformational changes in the binding site. The sequential conformational changes in amino acids will cause large movements in transmembrane helices.^15−17^ Throughout a series of conformational changes in GPCR, the receptor becomes activated, and then, messages of the ligand will be relayed to the cell.^18^ The signaling transition is defined as a sequence of conformational changes in the receptor preparing the intracellular region of it to be coupled with the G protein. However, any disruption in the signaling transition in GPCRs may cause some diseases; for example, it can amplify cancer progression.^10,19,20^ Due to the significance of the ligand-mediated signaling pathway, the GPCRs conformational changes have been the subject of remarkable academic efforts to understand diseases associated with the signaling networks.^11,21,22^ Undesired pathways in the GPCRs may also inhibit the accurate message-passing of the drugs into the cell. That will result in either side effects or low efficacy of the drugs targeting GPCRs. Experimental and theoretical methods (crystallography, NMR, single-molecule force spectroscopy, and spectroscopic methods such as FRET/BRET) are being used to investigate the GPCRs’ signaling mechanisms.^23,24^ By investigating the conformational changes in GPCRs within activation states, we can identify the transition pathways connecting the intermediate states with respect to activity levels of the receptor. However, the structural features of intermediate states that coordinate signaling pathways in GPCRs remain poorly understood. It is because of the time resolution required for resolving the dynamics of a protein both experimentally and computationally. Generally, experimental techniques can only provide a few snapshots of GPCR structures (normally active and inactive states) which is insufficient to map the signaling pathways connecting all states of activation. On the other hand, computational methods such as molecular dynamics (MD) simulations that can inform about dynamics of the proteins require significant statistical sampling and long trajectories. Obtaining sufficient trajectories for a complete GPCR activation process via MD is computationally expensive since it takes milliseconds to seconds.^25−27^ One solution is to run thousands of short simulations and extract knowledge from trajectory aggregation. Specifically, for GPCRs, multiple experimental crystal structures at different activity levels are available; therefore, different MD simulations could be initiated to collect robust statistics. With tremendous numbers of trajectories, a significant challenge arises from the high-dimensional data and interpretation. To interpret the high-dimensional data, machine learning (ML) models can be used to gain physical insight into the correlation between conformational structures of GPCRs and their activation states.^28−32^ In this study, we develop an ML model based on available experimental information on GPCRs to predict the activity level of a given GPCR structure. Using this model, we evaluate the activity levels of thousands of trajectories of β_2_*AR* receptors and predict the transition pathways between states of activation by ordering the activity levels corresponding to protein structural features. This is achieved by reaction coordinates correlated to the estimated activity levels. By taking advantage of our model, multiple intermediate states that give rise to activity levels of the receptors will be identified. In addition, using this method, the strength of contribution of each amino acid to the activity level can be assessed in order to highlight key residues driving the transition between activation states in GPCRs. Eventually, for the purpose of enhancing efficacy of drugs, we can design drugs that mainly target the principal amino acids in the receptors.

## Methods

**Figure 1:**
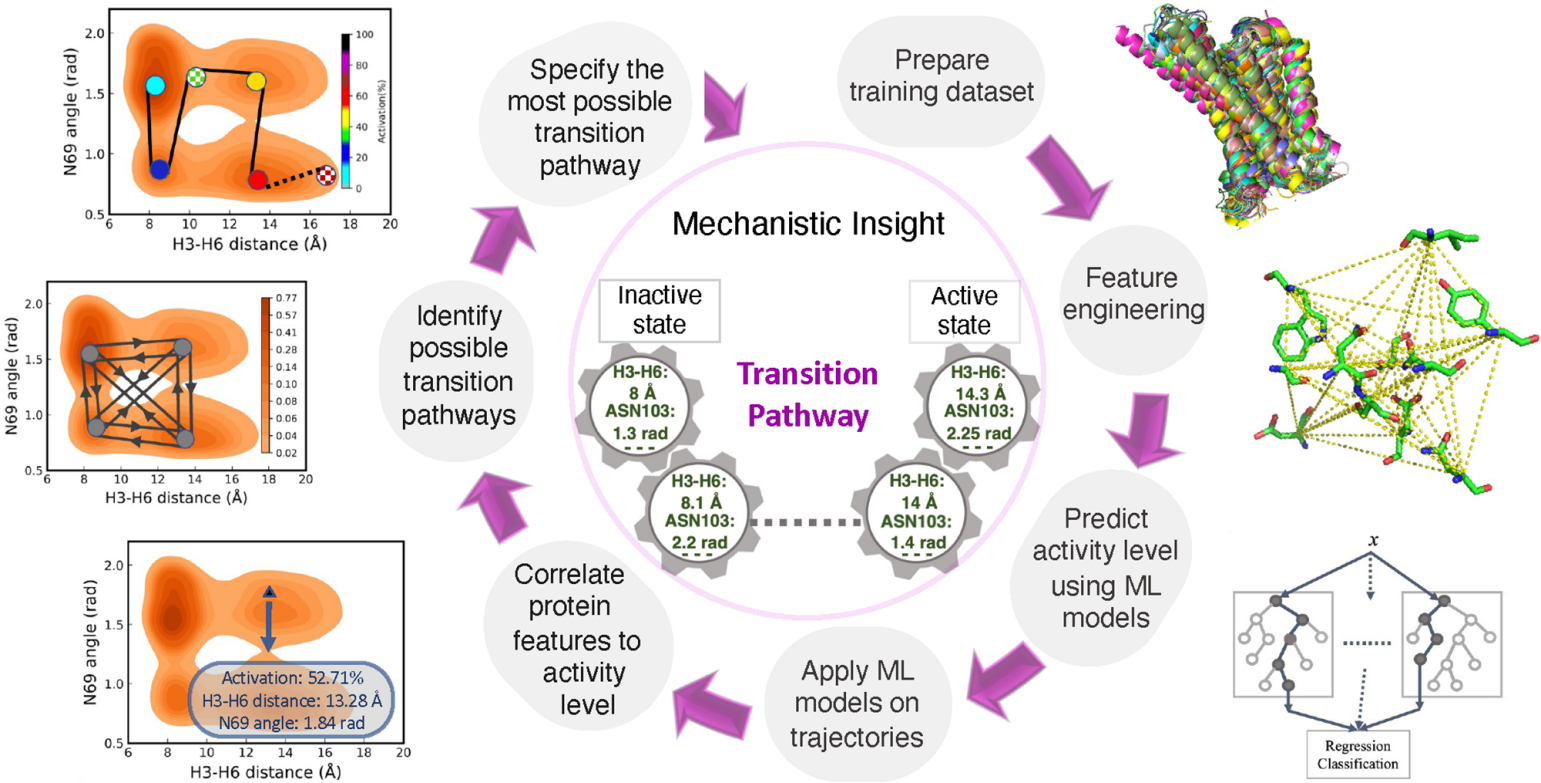
Framework of transition pathway identification between different activation states of a receptor. It consists of training set preparation, feature engineering, activity level prediction using shallow ML models, applying the most accurate ML model on MD trajectories, correlating protein structural features to the activity level, and finally identifying the most probable transition path between inactive, intermediate, and active states in GPCRs.

Figure 1 introduces the overall framework of our developed method. The stages of the method are as follows: we first prepared an appropriate experimental data set of receptors containing protein structure information, state of activation, and activity level corresponding to the conformational structure of the receptors. With this data set, we defined biophysics-aware features through feature engineering as input to train different shallow ML models.

### Data Set and Preprocessing

The data set we used for training ML models is taken from GPCRdb database.^33^ It provides experimental information on GPCRs such as ligand-binding constants, phylogenetic diagrams, protein binding, configuration, and signal transduction, as well as different categories of analysis tools and computational data such as homology models and multiple sequence alignments.^33,34^ The training data set includes 555 protein structures of GPCRs containing 105 unique receptor types^34^ (see Supporting Information). The PDB database contains three-dimensional structural data of proteins and drug molecules.^35^ The state of activation and activity level of the receptors are also obtained from the GPCRdb database.^33^ To prepare the data set for training ML models, we extract only structures of transmembrane (TM) domains since they play crucial roles in the activation process of GPCRs. The truncated structures of 555 proteins are then spatially aligned to ensure that amino acids located at the same positions are extracted from all the receptors for feature engineering (see Supporting Information).

### Feature Engineering

**Figure 2:**
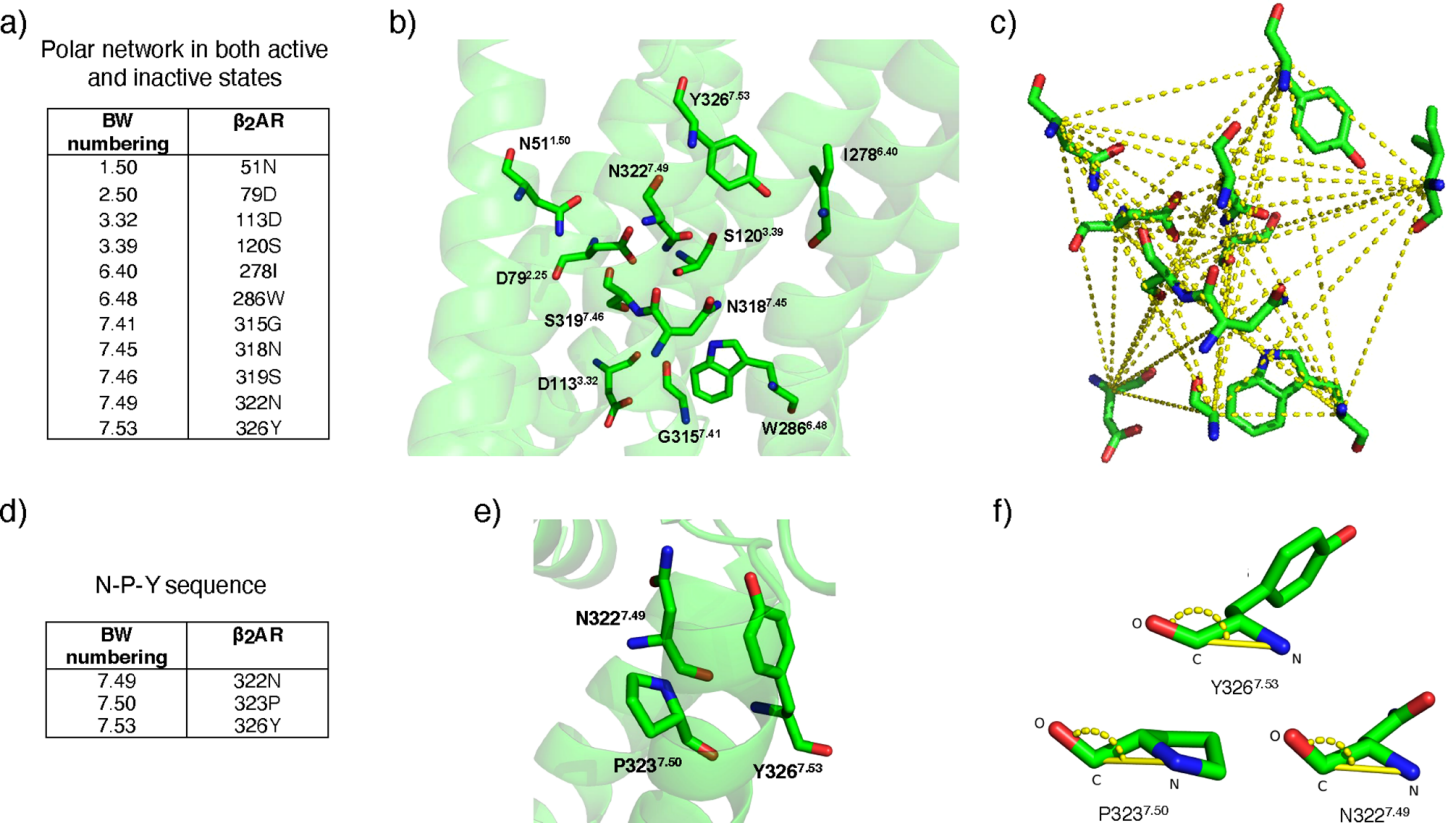
Feature engineering and the type of features selected for activity level prediction using ML. Ballesteros–Weinstein numbers are used to label the amino acids. (a) Amino acids engaged in the polar network in β_2_*AR* receptor. (b) Arrangement of polar network residues and their positions in the receptor. (c) Yellow dashed lines represent contact distances between amino acids in the polar network used as input to the ML models. (d) Amino acids involved in the NPxxY motif in the receptor. (e) Position of *N*322^7.49^, *P*323^7.50^, and *Y*326^7.53^ amino acids (as N, P, and Y residues in NPxxY motif) on the receptor. (f) Angle features between O–C–N atoms in the N, P, and Y residues used as input to the ML models. Both features are conserved in the active, intermediate, and inactive states of the β_2_*AR* receptor.

With the training data set, we are able to define a classification task to estimate the state of activation and a regression task to predict the activity level of a given receptor. In order to develop an ML model, we need to engineer biophysics-aware features (recent knowledge of GPCRs structural features to design these features with the goal of achieving higher prediction accuracy) from the data set to train ML models. Although all GPCRs possess seven-transmembrane structures, only a limited number of amino acids are conserved among them, meaning that amino acids with similar functions are located at the same positions in GPCRs. To train ML models, we included particular conserved features suggesting that they may have similar functions in the activation processes.^36^ The notion of biophysics-aware features refers to the features that are important on the activation paths of the GPCRs. These features were recognized by our literature survey. In this work, we focused on polar network and NPxxY motif contributing to GPCRs signal transduction (Figure 2).^37^ A polar network is a group of amino acids located mainly in the first, second, third, sixth, and seventh transmembrane domains^36^ (Figure 2a, b). The network includes the hydrogen bonds stabilizing the active and inactive states of GPCRs. Comparing active and inactive structures of GPCRs, the polar network has to be rearranged to achieve the active conformation.^36^ In our previous work,^31^ we trained ML models with features comprising contact distances between two amino acids in random 21 pairs of residues where at least one residue in each pair engaged in the polar network. With those features, ML models could provide activation prediction accuracy of 93.69% for classification tasks.^31^ However, since the main focus of this study is regression tasks (i.e activity level prediction), we fine-tuned different combinations of structural features to achieve higher accuracy. We found out that ML models trained with only conserved features can provide higher activation prediction accuracy. Hence, in this study, we measured the *C*_α_ contact distance between each pair of amino acids engaged in the polar network, as shown in Figure 2c (see Table S1 in Supporting Information). Moreover, we computed angular features in the NPxxY motif which is a conserved sequence of amino acids in the seventh transmembrane region^38^ (Figure 2d, e). The conserved NPxxY motif in GPCRs has been demonstrated to affect the activation process in the receptors, such as activation of phospholipase C,^39,40^ phospholipase D,^41^ adenylyl cyclase,^42^ and activation of Gq protein by cholecystokinin B receptor.^43^ It is studied that the NPxxY motif is necessary for internalization of the β_2_ receptor–adrenergic receptor.^44^ It is also investigated that NPxxY motif in formyl peptide receptor (FPR) plays a critical role in G protein coupling, phosphoinositide hydrolysis, MAP kinase activation, receptor internalization, and chemotaxis.^37^ For this sequence, we defined angle features between O–C–N atoms in *N*322^7.49^, *P*323^7.50^, and *Y*326^7.53^ residues involved in the NPxxY motif (Figure 2f). In total, we trained ML models with 58 features including 55 contact distances and three angle features. Some of the amino acids included in the features to train our ML model, highly interact with the ligand in the binding pocket. Such interactions are significantly essential for designing new ligand/drugs targeting GPCRs. For example, Jiménez-Rosés et al.^45^ illustrated ligands interactions with *W*^6.48^ residues and ionic interactions of *D*^3.32^ residue. Kooistra et al.^46,47^ studied conformational changes in the receptor binding site and the role of *D*^3.32^ residue to identify new ligands based on GPCRs structures. de Graaf et al.^48^ determined that the β-adrenoceptor and H1R pockets consist of 33 residues including *D*^3.32^, *W*^6.48^, and *N*^7.45^. These three residues are also part of the polar network utilized in our biophysics-aware features to train ML models.

### Structure–Activity ML Predictor

With respect to the size of the features computed in the previous section and the training data set, we benchmarked different shallow ML models to predict the activity level of a given receptor. We trained three shallow ML models including Decision Tree, Random Forest, and XGBoost. Random Forest is an ML method that contains decision trees, and output is the mode of trees, while the XGBoost model implements a gradient-boosted trees algorithm to minimize the loss.^49,50^ We used the scikit-learn package to benchmark shallow ML models.^51^ A 5-fold cross-validation is performed for these models for both classification and regression tasks. The ML classifiers are then applied to predict the activation state of receptors (in three classes: inactive, intermediate, and active states). The ML regressors are also implemented to predict the activity level of receptors (in the range of 0%–100%). Table 1 demonstrates performances of the ML models in the activation state and activity level prediction. The accuracy and mean absolute error (MAE) for classification and regression tasks are reported, respectively, as two metrics to estimate the performance of the ML models. The results reveal that the XGBoost model predicts both activation state and activity level of a given receptor with higher accuracy compared to the Decision Tree and Random Forest models (Table 1). We also computed the *R*^2^ (coefficient of determination) for the XGBoost model which is 0.99.

**Table 1:** Performances of XGBoost, Random Forest, and Decision Tree Models in Classification and Regression Tasks with Standard Deviations in Parenthesis.

ML Model	State prediction accuracy (%)	MAE for activation regression (%)
XGBoost	97.37 (0.02)	8.55 (0.67)
Random Forest	94.48 (0.02)	9.52 (0.81)
Decision Tree	90.45 (0.02)	18.05 (1.60)

## Results and Discussion

We applied the most accurate ML model (XGBoost) on MD trajectories in order to estimate the correlation between activity level and conformational state (structure) of receptors. The main goal, which is to interpret the transition pathway between active, intermediate, and inactive states of GPCRs, is achievable by ordering the activity levels corresponding to the structural properties of the receptors.

### Activity Levels of Trajectories

**Figure 3:**
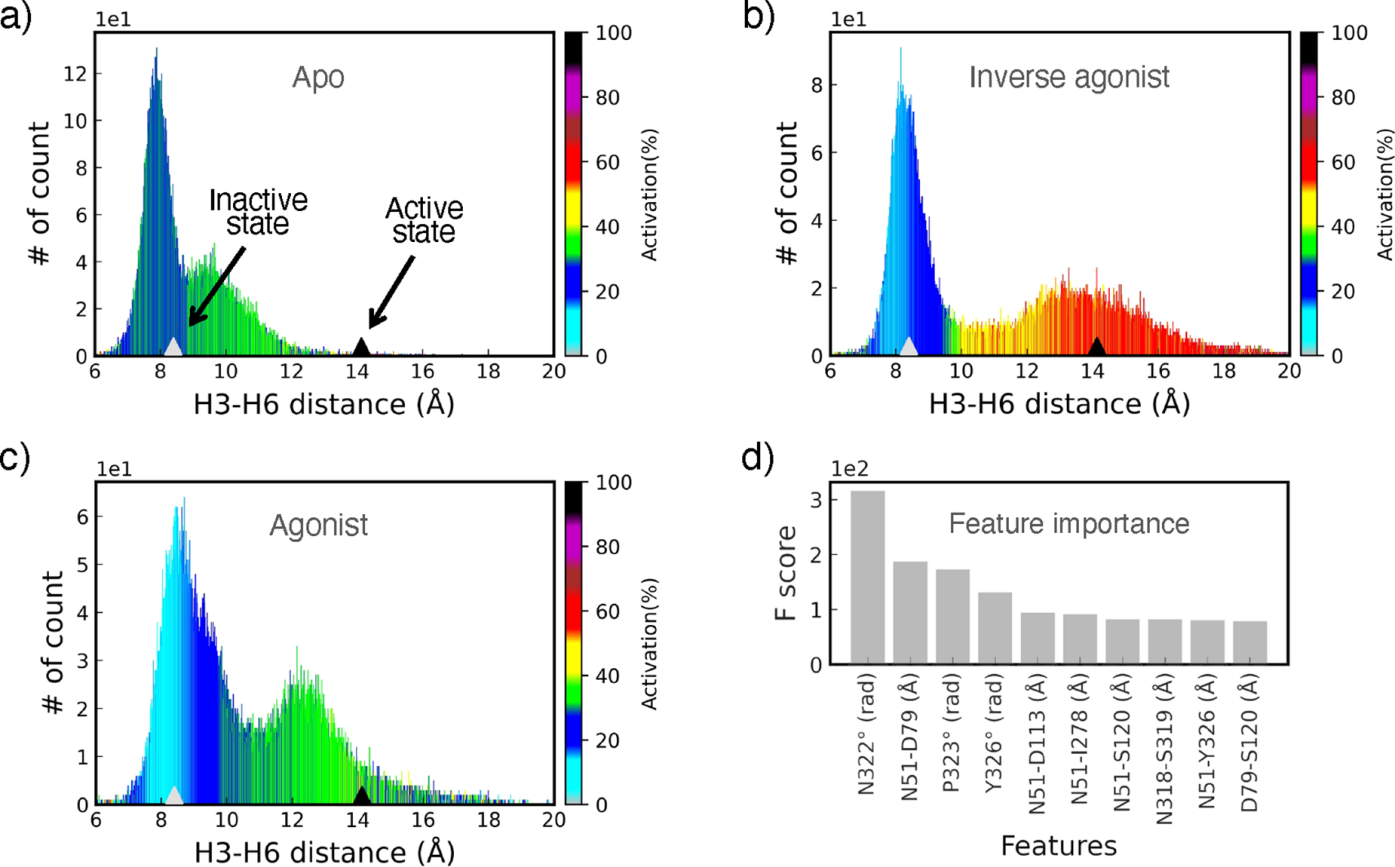
Histograms of H3–H6 distance for different conformational states of β_2_*AR* receptor. The color bars show the activity levels (%) predicted by the XGBoost model. The parameter used for generating the histograms is the *C*_α_ contact distance between *R*131^3.50^–*L*272^6.34^ residues. (a) Apo; the receptor without any ligand. (b) Inverse agonist; with inverse agonist carazolol bound to the receptor. (c) Agonist; with agonist BI-167107 coupled to the receptor. (d) Top ten features obtained via feature importance of the XGBoost model; note that the features showed with unit Å are *C*_α_ contact distances between the two residues and those with unit rad are angles between O–C–N atoms in the residues.

MD simulation provides insights into structural ensembles and dynamics of proteins.^52−56^ Using MD trajectories and XGBoost model, we can predict the activity level of each frame of the trajectory and build transition pathways between states of activation by correlating protein structural features to the activity. This can be achieved via statistical sampling and finding stable states. The activation and dynamic of the β_2_-adrenoceptor have been studied using ML in this work. This receptor plays a crucial role in inducing bronchodilation in patients suffering from asthma^57^ and chronic obstructive pulmonary disease (COPD).^58^ The conformations of β_2_*AR* have been heavily studied using computational and experimental methods to describe the significant structural features of the receptor correlated to activation states.^59^ In this study, we used MD simulation trajectories containing 2.15 ms of dynamics of ligand-free active (PDB 3P0G)^60^ and inactive (PDB 2RH1)^61^ structures of β_2_*AR*, as well as in the presence of two ligands; the partial inverse agonist carazolol and full agonist BI-167107.^17^ In each simulation, the protein was placed in a POPC lipid bilayer and solvated in TIP3P water molecules. The massive parallel simulations have been run on Google’s Exacycle platform.^62^ All simulations were performed using the Gromacs 4.5.3 MD package^63^ and carried out with a 2 fs time step. The trajectories were saved every 0.5 ns. For each data set including apo, agonist, and inverse agonist conformational structures of the β_2_*AR* receptor, we randomly chose 5000 MD trajectories (including ∼150,000 frames) (Figure 3) (see Supporting Information). By applying the ML model to the MD trajectories, we are able to interpret how the activity level of a receptor vary with regard to its dynamics. To apply the XGBoost model on every single frame of the trajectory, we first prepared the input to the ML model (as explained in the feature engineering section) for each frame and then used the ML model to predict the corresponding activity level. It is necessary to present predicted activity levels of the trajectories in terms of a known structural feature of GPCRs. One of the experimentally best-known features of GPCRs related to activation states is the Helix3–Helix6 (H3–H6) distance. It is notable that the H3–H6 distances in the inactive and active structures of β_2_*AR* receptor are ∼8.4 and ∼14.1 Å, respectively. Since the activity level is highly correlated with the H3–H6 distance,^64^ the feature is not used to train ML models. This gives us the opportunity to validate the activity level predicted by the ML model using its correlation with the well-known H3–H6 distance feature. To investigate how the activity levels change in terms of a protein structural feature, we plotted related density histograms in different conformational states of the receptor mapped on corresponding predicted activity levels (Figure 3). For the density histograms in Figure 3, we measured the H3–H6 distance as the *C*_α_ contact distance between *R*131^3.50^–*L*272^6.34^ amino acids at each frame of the simulations. On the histograms, each bar is colored based on the average of predicted activities of data points associated with it. All figures showing the density histogram generated by the H3–H6 distance contain two points (shown by triangle features) introducing the experimental H3–H6 distance in the active and inactive crystal structures of the β_2_*AR* receptor. These two points are used to validate the performance of our model. With respect to these two points, we observe that the ML model predicted inactive, active, and intermediate states mostly around ∼8.4 and ∼14.1Å and between ∼8.4 and 14.1 Å, respectively. This result reveals that the ML predictor is accurate enough for both the classification and regression tasks. Based on the feature importance shown in Figure 3d, we can identify that the angular features of N322, P323, and Y326 involved in the NPxxY motif and N51 residue engaged in the polar network (five out of seven contact distances in the feature importance contain this residue) play critical roles in the activity level prediction. In addition, using Figure 3b containing the inverse agonist data set of the β_2_*AR* receptor, we investigated the relationship between the H3–H6 distance and the activity level of the receptor. Figure S4shows that in the inactive state, the activity level is correlated to the H3–H6 distance much stronger than in the active or intermediate states, meaning that when the receptor is in the inactive state a slight change in the H3–H6 distance can significantly affect the activity level (see Supporting Information).

### Identifying the Most Probable Transition Pathway

**Figure 4:**
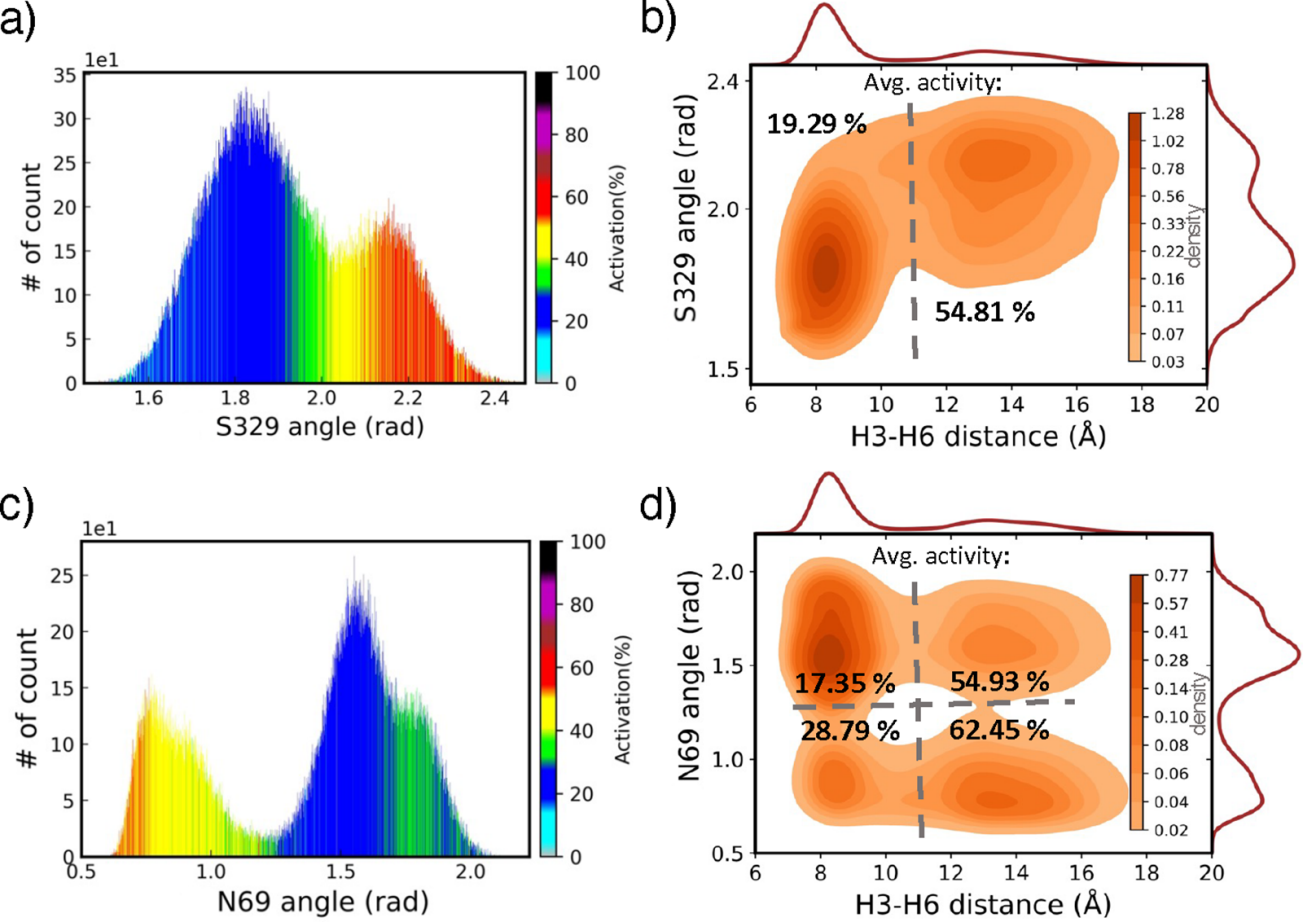
1D and 2D density histograms corresponding to activity level predictions in the inverse agonist data set of β_2_*AR* receptor projected on two reaction coordinate features. (a) 1D density histogram projected on *S*329^8.47^ residue angle feature. (b) Density landscape of *S*329^8.47^ residue angle feature vs H3–H6 distance with average of activation denoted for two regions separated by the dashed line. (c) 1D density histogram with predicted activity levels generated by angle feature of *N*69^2.40^ residue. (d) Density landscape of *N*69^2.40^ residue angle feature vs H3–H6 distance with average of activation computed for four regions separated by the dashed line. The color bars in (a) and (c) represent the activity level predicted by the XGBoost model, and the color bars in (b) and (d) show the density of states.

**Figure 5:**
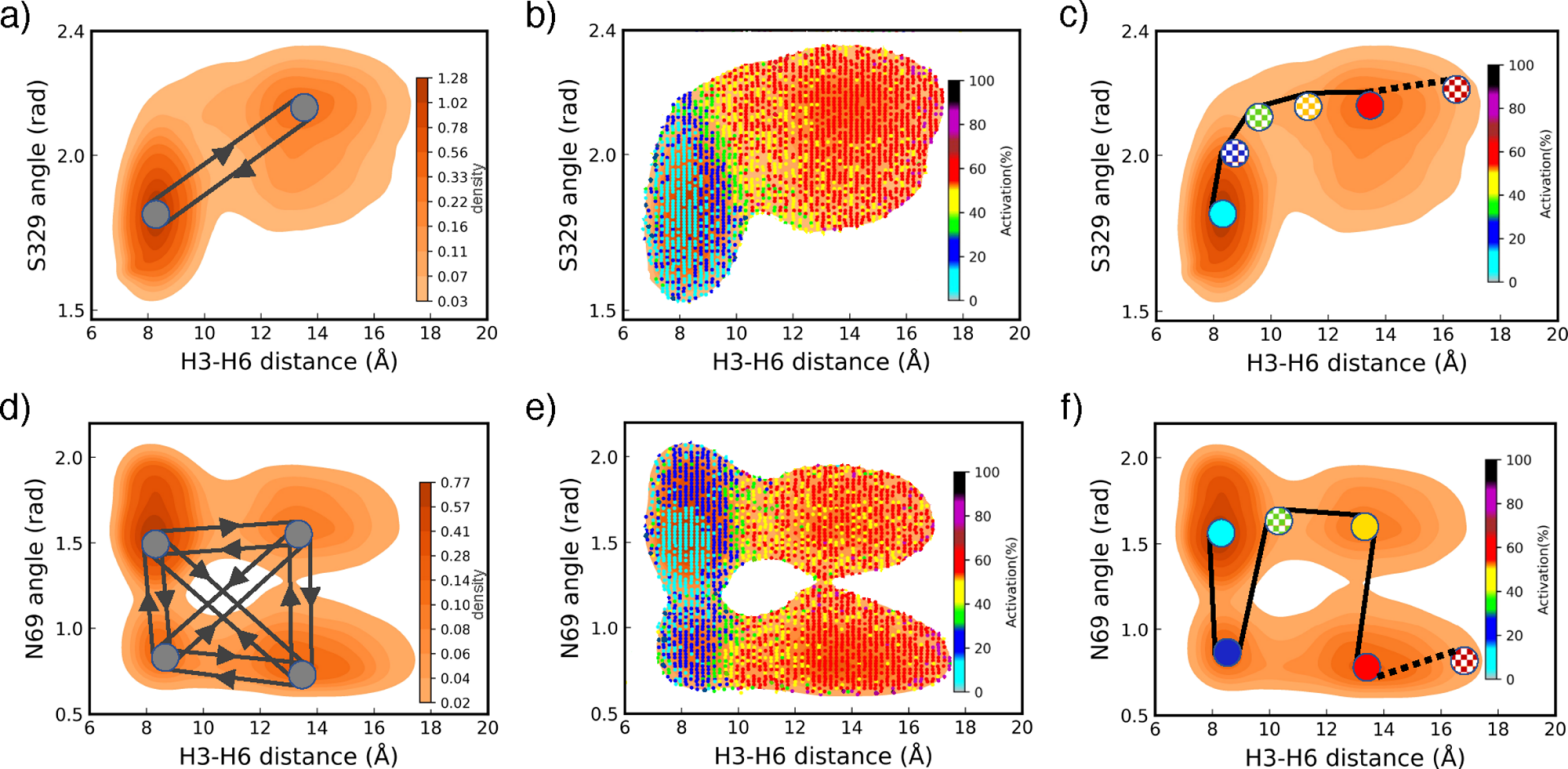
Density landscape of β_2_*AR* receptor projected on H3–H6 distance and angle features with activity levels. (a) Density landscape of *S*329^8.47^ angle feature vs H3–H6 distance with two possible pathways based on the density peaks. (b) Predicted activity levels on density landscape of *S*329^8.47^ angle feature and H3–H6 distance at small areas (3 × 10^–3^ rad.Å). (c) Proposed activation pathway for the two features (*S*329^8.47^ angle feature and H3–H6 distance) with a gradual change in the activity levels shown by filled and patterned circles. (d) Few potential transition pathways between four states populated with density peaks of *N*69^2.40^ angle feature and H3–H6 distance. (e) Predicted activity levels for density landscape of *N*69^2.40^ angle feature and H3–H6 distance at the small areas (3 × 10^–3^ rad.Å). (f) Proposed activation pathway for the two features (*N*69^2.40^ angle feature and H3–H6 distance) with a gradual change in the activity levels shown by the filled and patterned circles. The filled circles (in (c) and (f)) present the states recognized by the high-density peaks while the patterned filled circles are samples showing the activity levels on the pathways between the density peaks.

**Figure 6:**
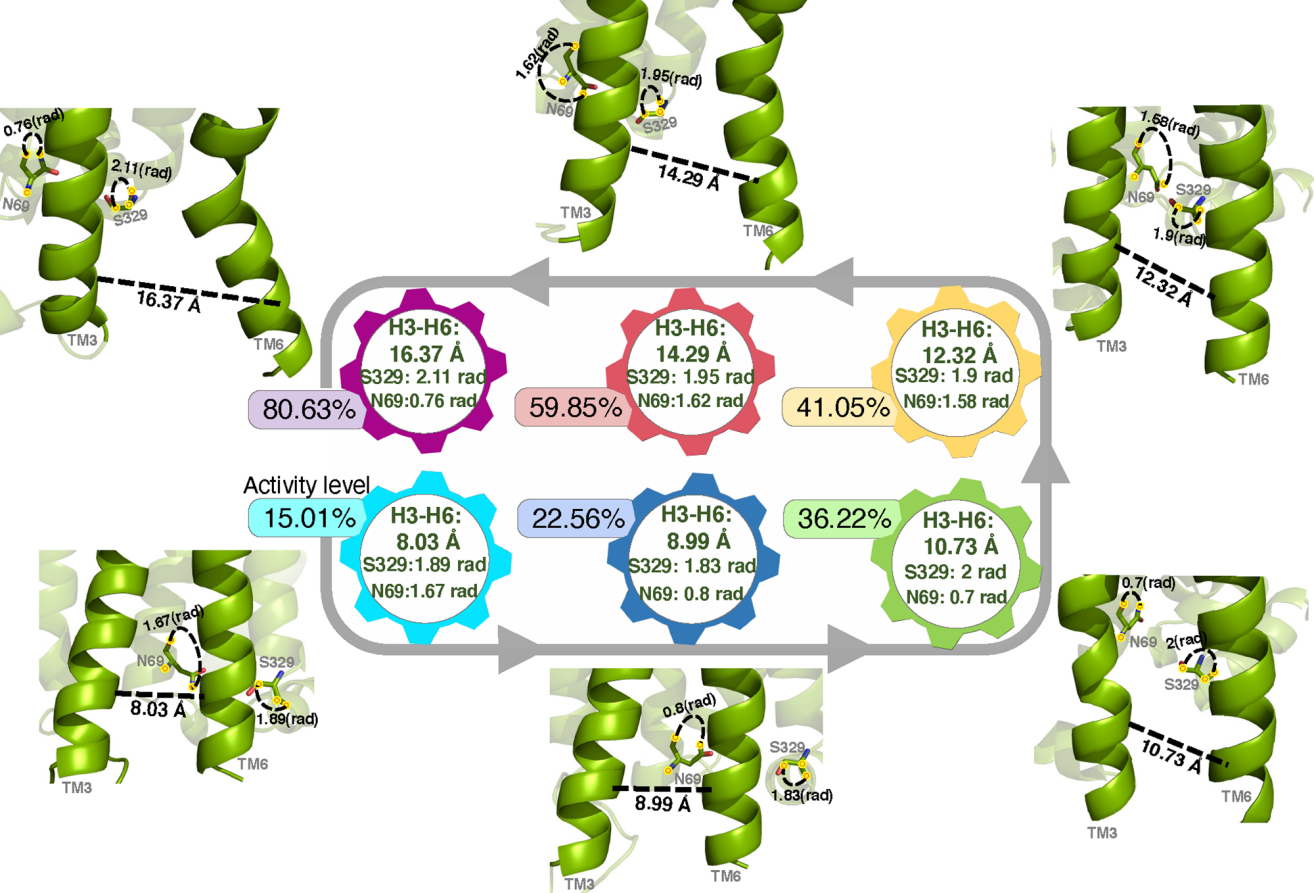
Structure and activity level demonstration of transition states for few structural features in β_2_*AR* receptor (H3–H6 distance, *S*329^8.47^ angle feature, and *N*69^2.40^ angle feature) ranked in ascending order of activity levels.

In order to identify the complete transition pathway between states of activation, the correlations between the activity level and all structural features of a receptor (for example, angular changes in each amino acid or residue pairwise distances) are essentially required. This will provide an n-dimensional density landscape mapped on predicted activity levels. However, to be able to visualize such a correlation, we projected 2D density maps using two structural features of the β_2_*AR* receptor in the inverse agonist data set. For all the 2D plots, one dimension (on the horizontal axis) is H3–H6 distance. Figure 4a represents the histogram of the *S*329^8.47^ angle feature, and its combination with the histogram of the H3–H6 distance provides a density landscape as shown in Figure 4b. Figure 4c and d illustrate the density projection of the *N*69^2.40^ angle feature and density landscape corresponding to the feature and H3–H6 distance, respectively. Figure 4b and d also introduce the average of activity levels in each distinct region in the landscapes separated by dashed lines. The density landscapes verify that the distributions of densities vary with respect to the protein features and activity levels, meaning that each region of density has its own majority of activation state. For example, Figure 4b shows that going from one peak of density at the H3–H6 distance of ∼8 Å to another peak at ∼14 Å both of the averages of activity levels and the *S*329^8.47^ angle feature increase (average of activity level increases from 19.29% to 54.81% and *S*329^8.47^ angle feature from 1.8 to 2.2 rad). However, such changes are more complicated in the density landscapes including the *N*69^2.40^ angle feature and H3–H6 distance, as shown in Figure 4d. It can be shown in Figure 5a and d that without using ML models, there are multiple potential pathways connecting the peaks of densities in different directions (shown with gray circles and arrows). We followed three steps to find the most probable pathway: (1) Find all the high-density peaks over the landscape and connect them (two by two) through the minimum-density paths (Figure 5a, d). (2) Predict the average activity levels all over the landscape. Here, we measured the average of activity levels in small areas 3 × 10^–3^ rad.Å (Figure 5b, e). (3) Connect the states in ascending order of activation in order to build the transition pathway from inactive to active states (Figure 5c, f). Therefore, the combination of density of states and activity levels can yield the most probable pathways between activation states of a receptor. As shown in a 3D density representation in Figure S2 (including *S*329^8.47^ angle, *N*69^2.40^ angle, and H3–H6 distance), the active and inactive states are separately clustered in the z-direction (see Supporting Information). However, it is difficult to visualize the transition pathway (similar to the pathways shown in Figure 5c, f) in such 3D plots. Hence, for visualization purposes, we performed transition pathway identification only for two features here. However, it should be done in high dimensional space (n-dimension corresponding to n-structural features of the receptor). Then, we can identify the global most possible transition pathway between activation states of the receptor by ranking all states in terms of activity levels. Additionally, it can be observed in Figure 5 that the H3–H6 distance and *S*329^8.47^ angle feature are increasing as the activation increases, while the *N*69^2.40^ angle feature is oscillating for similar changes in activation. Therefore, our framework not only is capable of identifying the path of transition between activation states but also can evaluate the strength of contribution of each protein structural feature to the activity level. This provides significant mechanistic insight into GPCRs activation and drug discovery. By estimating the influence of each amino acid in the activation process, we can design drugs that mainly target the principal amino acids driving the transition pathway between activation states. In that way, we can minimize side effects or maximize efficacy of the drugs targeting GPCRs. Figure 6 shows a few pieces of a possible transition pathway between the activation states in the β_2_*AR* receptor containing the H3–H6 distance, *S*329^8.47^ angle feature, and *N*69^2.40^ angle feature.

## Conclusion

In this work, we have developed an ML-based framework to predict the most probable transition pathway between activation states of GPCRs based on structural features of the receptors. To achieve this, we devised an ML model to predict the activity level of GPCRs. We considered two conserved biophysics-aware features containing the polar network and NPxxY motif to train shallow ML models. The XGBoost model can be used to predict activation state and activity level of GPCRs with 97.37% accuracy and 8.55% MAE, respectively. We then applied the ML model to MD trajectories of the β_2_*AR* receptor to correlate protein structural dynamics to the activity levels with the goal of finding the most probable transition pathway between states of activation. By combining density of states and the activity levels, we were able to find the most probable pathway. In this study, we featurized two residues (for easy visualization) in all the trajectories and then projected their density landscapes providing many possible transition pathways between density peaks. To identify the most probable pathway, we evaluated the activity levels in all the states over the landscape and then ranked the activity levels correlated to the defined structural features in ascending order to identify the path of transition from inactive to active states. The complete transition pathway can be predicted by considering all n-structural features of the receptor. Overall, this framework provides valuable knowledge on the activation mechanism of GPCRs and can significantly accelerate the discovery of drugs targeting GPCRs.

## Data Availability

All the information containing the processed PDB structures of the GPCRs, the classification and regression labels of the receptors, and all the scripts for training and testing ML models used in this study are available here: https://github.com/pmollaei/GPCRpath.

## Supporting Information Available

(Preprocessed GPCR structures, list of residue pairs in polar network used as input for ML models, 3D visualization of three structural sample features used in this study, minimum length of trajectories required to reproduce this study in other types of GPCRs, and activation level changes with respect to H3–H6 distance in different activation states of β_2_*AR* receptor. PDF)
