# Supplementary material for: Activity Map and Transition Pathways of G Protein-Coupled Receptor Revealed by Machine Learning
Source: J Chem Inf Model. 2023 Apr 10;63(8):2296–304. doi: 10.1021/acs.jcim.3c00032 (PMC10131220; doi:10.1021/acs.jcim.3c00032)
Supplement: Supplementary file 1 — ci3c00032_si_001.pdf [file ci3c00032_si_001.pdf]

Supporting Information: Activity Map and Transition Pathways of G Protein Coupled Receptor Revealed by Machine Learning

Parisa Mollaei[†] and Amir Barati Farimani[*,‡,¶,§]

†*Department of Mechanical Engineering, Carnegie Mellon University, 15213,USA*

‡*Department of Mechanical Engineering, Carnegie Mellon University, 15213, USA*

¶*Department of Biomedical Engineering, Carnegie Mellon University, 15213, USA*

§*Machine Learning Department, Carnegie Mellon University, 15213, USA*

E-mail: barati@cmu.edu

1 GPCRs structure data preprocessing

The training dataset contains 555 proteins from the RCSB server. First, we aligned all these proteins since the features we defined for training ML models are position-dependent and include contact distances of residues engaged in the polar network and angle features of residues involved in the NPxxY motif. To overcome the challenge of feature extraction, we spatially aligned all the GPCRs to a reference receptor (Fig.S1c). The reference receptor is the Neurotensin Receptor1 (NTSR1, PDB:6UP7) which belongs to Class-A (Rhodopsin) GPCR. All the proteins are aligned by using the align tool of PyMOL software.[1,2] For this study, we only selected amino acids that are on the helical structures and removed all the

intracellular loops. This ensures that only the transmembrane domains having significant roles in the activation process are used for extracting features to train ML models.

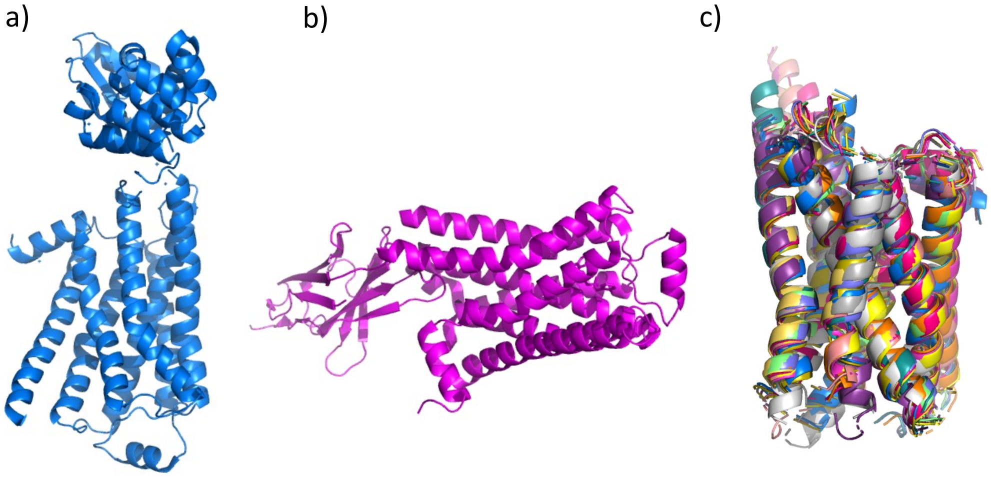

Figure S1: The visualization of the original (a) inactive and (b) active structures of $\beta_2 AR$ receptor along with TM region and other sub-units. (c) some transmembrane domains of GPCRs structures in the training dataset aligned with the NTSR1 receptor (PDB: 6UP7).

2 Residue-pairs in the polar network

Table S1 represents all 55 pairs of residues in the polar network of $\beta_2 AR$ receptor. The C_α contact distance between each of the residue-pairs is measured as input to the ML models.[3] This list is conserved in the active, intermediate, and inactive conformations of $\beta_2 AR$ receptor.

Table S1: List of 55 residue-pairs in polar network of $\beta_2 AR$ receptor. The C_α contact distance between each residue pair in this list is measured for 555 proteins in the training dataset to train ML models.

$N51^{1.50}$-$D79^{2.50}$	$N51^{1.50}$-$D113^{3.32}$	$N51^{1.50}$-$S120^{3.39}$	$N51^{1.50}$-$I278^{6.40}$
$N51^{1.50}$-$G315^{7.41}$	$N51^{1.50}$-$N318^{7.45}$	$N51^{1.50}$-$S319^{7.46}$	$N51^{1.50}$-$N322^{7.49}$
$D79^{2.50}$-$D113^{3.32}$	$D79^{2.50}$-$S120^{3.39}$	$D79^{2.50}$-$I278^{6.40}$	$D79^{2.50}$-$W286^{6.48}$
$D79^{2.50}$-$N318^{7.45}$	$D79^{2.50}$-$S319^{7.46}$	$D79^{2.50}$-$N322^{7.49}$	$D79^{2.50}$-$Y326^{7.53}$
$D113^{3.32}$-$I278^{6.40}$	$D113^{3.32}$-$W286^{6.48}$	$D113^{3.32}$-$G315^{7.41}$	$D113^{3.32}$-$N318^{7.45}$
$D113^{3.32}$-$N322^{7.49}$	$D113^{3.32}$-$Y326^{7.53}$	$S120^{3.39}$-$I278^{6.40}$	$S120^{3.39}$-$W286^{6.48}$
$S120^{3.39}$-$N318^{7.45}$	$S120^{3.39}$-$S319^{7.46}$	$S120^{3.39}$-$N322^{7.49}$	$S120^{3.39}$-$Y326^{7.53}$
$I278^{6.40}$-$G315^{7.41}$	$I278^{6.40}$-$N318^{7.45}$	$I278^{6.40}$-$S319^{7.46}$	$I278^{6.40}$-$N322^{7.49}$
$W286^{6.48}$-$G315^{7.41}$	$W286^{6.48}$-$N318^{7.45}$	$W286^{6.48}$-$S319^{7.46}$	$W286^{6.48}$-$N322^{7.49}$
$G315^{7.41}$-$N318^{7.45}$	$G315^{7.41}$-$S319^{7.46}$	$G315^{7.41}$-$N322^{7.49}$	$G315^{7.41}$-$Y326^{7.53}$
$N318^{7.45}$-$N322^{7.49}$	$N318^{7.45}$-$Y326^{7.53}$	$S319^{7.46}$-$N322^{7.49}$	$S319^{7.46}$-$Y326^{7.53}$
$N51^{1.50}$-$W286^{6.48}$	$N51^{1.50}$-$Y326^{7.53}$	$D79^{2.50}$-$G315^{7.41}$	$D113^{3.32}$-$S120^{3.39}$
$D113^{3.32}$-$S319^{7.46}$	$S120^{3.39}$-$G315^{7.41}$	$I278^{6.40}$-$W286^{6.48}$	$I278^{6.40}$-$Y326^{7.53}$
$W286^{6.48}$-$Y326^{7.53}$	$N318^{7.45}$-$S319^{7.46}$	$N322^{7.49}$-$Y326^{7.53}$	

3 3D visualization of three features

For visualization purposes only 2D density landscapes are represented in Fig.5. However, to identify complete transition pathways between activation states of GPCRs, all structural features of the receptors must be included in the results that will result in n-dimensional density. Fig.S2 shows the 3D density of S329 angle, N69 angle, and H3-H6 distance features (included in Fig.5) correlated to activity levels. It is obvious that the active and inactive states are separately clustered in z-direction (based on H3-H6 distances), however, it is difficult to visualize transition pathways (as shown in Figure 5c,f) in such 3D plots.

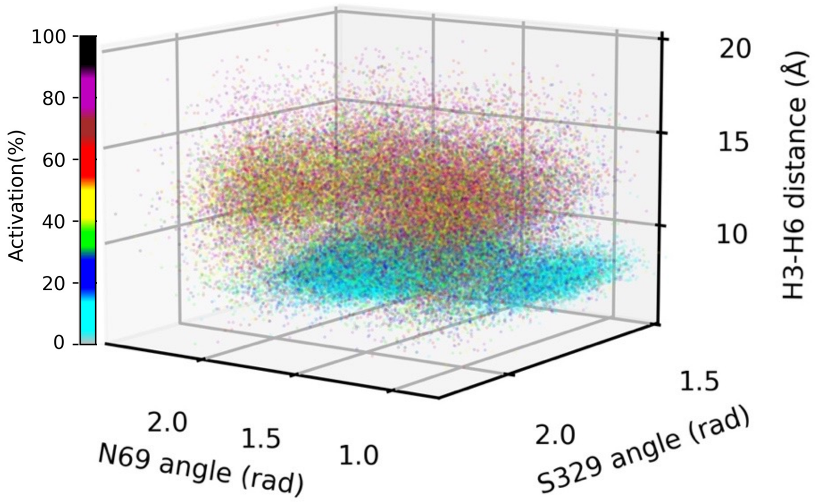

Figure S2: 3D density of S329 angle, N69 angle, and H3-H6 distance (the features included in Fig.5) correlated to the activity levels predicted by the XGBoost model.

4 Minimum length of trajectories required to reproduce this study in other GPCR

In this study the length of trajectories varied, however, in order to identify the minimum length of trajectories (total number of frames of trajectories) required to reproduce this study in other GPCRs, we have defined bin ratio:

$$\text{bin ratio} = \frac{\text{number of bins satisfy the required activity levels}}{\text{total number of bins}}$$

The bin ratio must be higher than 50% for all activation states to reproduce this study. Fig.S3 presents the bin ratio for different numbers of frames of trajectories (lengths of 100, 1000, 10000, and 100000 frames) of $\beta_2 AR$ receptor. It can be observed from the plots that bin ratio higher than 50% for all activation states is required to reproduce the results of this study.

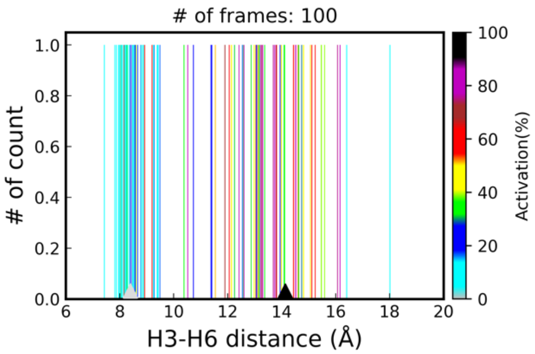

Bin ratio in Inactive state domain: 73%
Bin ratio in Active state domain: 27%
Bin ratio in Intermediate state domain: 46%

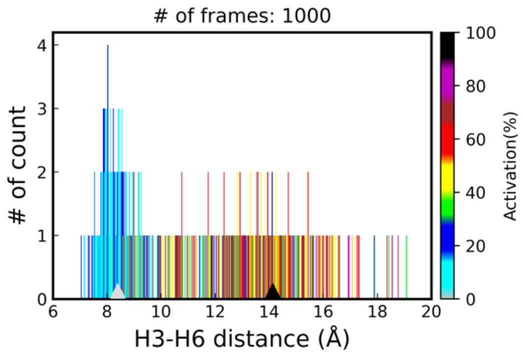

Bin ratio in Inactive state domain: 76%
Bin ratio in Active state domain: 43%
Bin ratio in Intermediate state domain: 48%

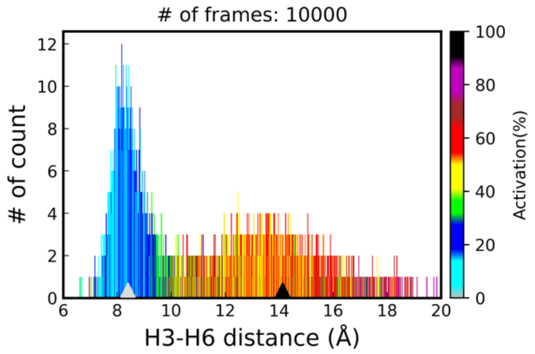

Bin ratio in Inactive state domain: 78%
Bin ratio in Active state domain: 46%
Bin ratio in Intermediate state domain: 48%

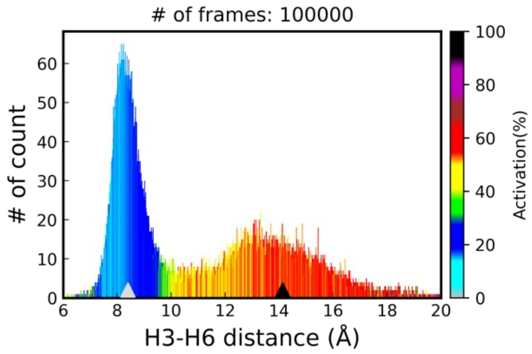

Bin ratio in Inactive state domain: 85%
Bin ratio in Active state domain: 57%
Bin ratio in Intermediate state domain: 62%

Figure S3: Bin ratio in the inactive, active, and intermediate states corresponding to different lengths of trajectories in the inverse agonist dataset of $\beta_2 AR$ receptor

5 Activity level changes with respect to H3-H6 distance in transition between states of $\beta_2 AR$ receptor

Fig.S4 shows the histogram of $\beta_2 AR$ receptor generated by H3-H6 distance for the inverse agonist dataset mapped on activity levels. The piece-wise linear slopes illustrate how the activity levels change with respect to the H3-H6 distance. As shown in the plot, the slope of activity level in terms of H3-H6 distance in the inactive states is much higher compared

to the active or intermediate states.

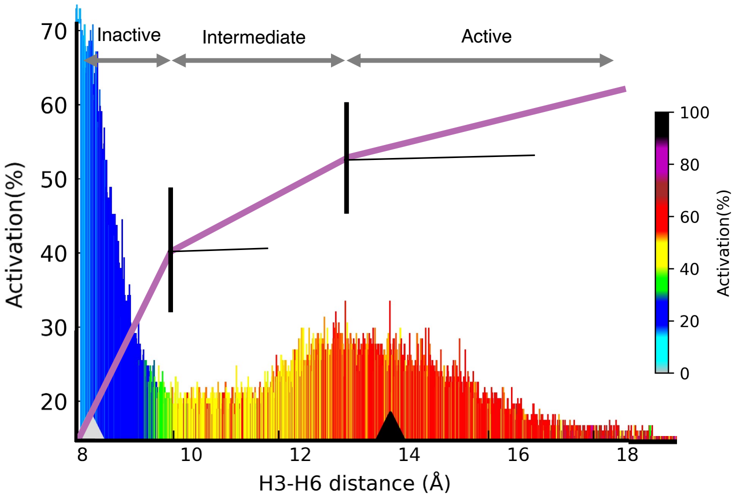

Figure S4: Different rates of activity with respect to H3-H6 distance in the inverse agonist dataset of $\beta_2 AR$ receptor. The black triangle on the x-axis represents the H3-H6 distance in the crystal active structure of $\beta_2 AR$ receptor and the grey one shows the H3-H6 distance in the crystal inactive structure of the receptor. The colorbar shows the predicted activity levels by XGBoost model (%0-%100)

References

(1) Schrödinger, LLC, The PyMOL Molecular Graphics System, Version 1.8. **2015**,

(2) DeLano, W. L., et al. Pymol: An open-source molecular graphics tool. *CCP4 Newsletter on protein crystallography* **2002**, *40*, 82–92.

(3) Huang, W.; Manglik, A.; Venkatakrishnan, A.; Laeremans, T.; Feinberg, E. N.; Sanborn, A. L.; Kato, H. E.; Livingston, K. E.; Thorsen, T. S.; Kling, R. C., et al. Structural insights into μ-opioid receptor activation. *Nature* **2015**, *524*, 315–321.
